# TNF-α Inhibitors in Combination with MTX Reduce Circulating Levels of Heparan Sulfate/Heparin and Endothelial Dysfunction Biomarkers (sVCAM-1, MCP-1, MMP-9 and ADMA) in Women with Rheumatoid Arthritis

**DOI:** 10.3390/jcm11144213

**Published:** 2022-07-20

**Authors:** Anna Szeremeta, Agnieszka Jura-Półtorak, Aleksandra Zoń-Giebel, Krystyna Olczyk, Katarzyna Komosińska-Vassev

**Affiliations:** 1 Department of Clinical Chemistry and Laboratory Diagnostics, Faculty of Pharmaceutical Sciences in Sosnowiec, Medical University of Silesia, Jedności 8, 41-200 Sosnowiec, Poland; ajura@sum.edu.pl (A.J.-P.); olczyk@sum.edu.pl (K.O.); kvassev@sum.edu.pl (K.K.-V.); 2 Department of Rheumatology and Rehabilitation, Specialty Hospital No. 1, Żeromskiego 7, 41-902 Bytom, Poland; azongiebel@gmail.com

**Keywords:** heparan sulfate/heparin, sVCAM-1, MCP-1, MMP-9, ADMA, rheumatoid arthritis, anti-TNF-α treatment

## Abstract

Sulfated glycosaminoglycans (sGAGs) are likely to play an important role in the development and progression of rheumatoid arthritis (RA)-associated atherosclerosis. The present study investigated the effect of anti-tumor necrosis factor-α (anti-TNF-α) therapy in combination with methotrexate on plasma sGAG levels and serum markers of endothelial dysfunction. Among sGAG types, plasma chondroitin/dermatan sulfate (CS/DS) and heparan sulfate/heparin (HS/H) were characterized using electrophoretic fractionation. Serum levels of soluble vascular cell adhesion molecule-1 (sVCAM-1), monocyte chemoattractant protein-1 (MCP-1), matrix metalloproteinase-9 (MMP-9) and asymmetric dimethylarginine (ADMA) were measured by immunoassays. The measurements were carried out four times: at baseline and after 3, 9 and 15 months of anti-TNF-α therapy. All analyzed parameters, excluding ADMA, were significantly elevated in patients with RA before the implementation of biological therapy compared to healthy subjects. Performed anti-TNF-α treatment led to a successive decrease in HS/H levels toward normal values, without any effect on CS/DS levels in female RA patients. The treatment was also effective at lowering the serum levels of sVCAM-1, MCP-1, MMP-9 and ADMA. Moreover, a significant positive correlation was found between the circulating HS/H and the 28 joint disease activity score based on the erythrocyte sedimentation rate (DAS28-ESR, r = 0.408; *p* <0.05), MCP-1 (r = 0.398; *p* <0.05) and ADMA (r = 0.396; *p* <0.05) in patients before the first dose of TNF-α inhibitor. In conclusion, a beneficial effect of anti-TNF-α therapy on cell-surface heparan sulfate proteoglycans (HSPGs)/HS turnover and endothelial dysfunction was observed in this study. This was manifested by a decrease in blood HS/H levels and markers of endothelial activation, respectively. Moreover, the decrease in the concentration of HS/H in the blood of patients during treatment, progressing with the decline in disease activity, indicates that the plasma HS/H profile may be useful for monitoring the efficacy of anti-TNF-α treatment in patients with RA.

## 1. Introduction

Rheumatoid arthritis (RA) is a systemic autoimmune connective tissue disease and the most common inflammatory arthritis, affecting up to 1% of the world’s population at any age. The peak incidence is between the ages 50 and 60, and women are two to three times more likely to develop RA than men [1,2,3]. Although chronic symmetric synovitis is a pathological hallmark of RA, numerous of extra-articular manifestations (EAMs) may occur, affecting the skin, eyes, heart and other organs, and thereby worsening the disease prognosis [3,4,5]. RA is associated with increased cardiovascular morbidity and mortality, mainly attributed to an accelerated atherosclerosis process. Compared to age-matched healthy subjects, patients with RA have twice the relative risk of myocardial infarction (MI) and up to a 50% increased cardiovascular disease (CVD) mortality risk [1,4,6]. Despite many studies, the exact cause of accelerated atherosclerosis accompanying RA is still not fully understood, although it may be associated with systemic inflammation and its interplay with several traditional (i.e., obesity, dyslipidemia, insulin resistance, hypertension, advanced age, male gender, physical inactivity and smoking) and new, non-traditional (i.e., genetic polymorphisms, autoantibodies, pharmacotherapy, duration of RA, high disease activity and EAM) cardiovascular risk factors. Among the above-mentioned mechanisms, chronic inflammation with immune dysregulation appears a crucial contributor to endothelial dysfunction during RA [1,4,7,8,9,10,11]. One of the main proinflammatory cytokines is tumor necrosis factor-alpha (TNF-α), which plays a central role in the pathogenesis of RA and is also implicated in all stages of atherosclerotic plaque development from its initiation to progression. Several TNF-α-mediated mechanisms may cause endothelial dysfunction in RA [9,12,13]. Firstly, TNF-α promotes the interaction between circulating leukocytes and endothelial cells (ECs) by increasing the expression of endothelial adhesion molecules such as vascular cell adhesion molecule-1 (VCAM-1) and intercellular adhesion molecule (ICAM-1), and various inflammatory cytokines. The continuous release of cytokines such as monocyte chemoattractant protein-1 (MCP-1) by activated ECs, T-cells and foam cells not only perpetuates inflammation and lipid accumulation into the subendothelial space but also affects vascular smooth muscle cells’ (SMCs’) activity [8,12,13]. Secondly, TNF-α induces impairment of endothelial-dependent vasodilation by increasing oxidative stress and reducing nitric oxide (NO) release. TNF-α is known to reduce the half-life of mRNA encoding endothelial nitric oxide synthase (eNOS) and contribute to the formation of asymmetric dimethylarginine (ADMA), which increases NO deficiency [9,10,11,12,14]. Finally, TNF-α increases platelet aggregation and reduces plasma fibrinolytic activity [15]. More precisely, TNF-α induces the expression of tissue factor and suppresses thrombomodulin, as well as the endothelial cell protein C receptor. It also promotes oxidative modifications of low-density lipoproteins (LDL) and induces the expression of matrix metalloproteinases (MMPs) in inflammatory cells and SMCs [9,11,12,16]. This leads to a loss of vascular elasticity, the formation of atherosclerotic plaques and their destabilization, and thus to an increased tendency of patients with RA to develop CVD [16].

Quantitative changes in the composition and structure of the extracellular matrix (ECM) components, especially proteoglycans (PGs) and their sugar constituents, sulfated glycosaminoglycans (sGAGs), are the integral parts of chronic inflammation during RA [17,18,19,20,21,22]. Sulfated GAGs are a family of linear unbranched heteropolysaccharides consisting of repeating disaccharide units of *N*-acetylated hexosamine and uronic acid or galactose. Since most of their sugars have sulfate or carboxyl groups, GAGs are highly negatively charged molecules. According to the disaccharide composition and sulfation pattern, GAGs are classified as the galactosaminoglycans, represented by chondroitin sulfate (CS) and dermatan sulfate (DS), and the glucosaminoglycans, with heparan sulfate (HS), heparin (H) and keratan sulfate (KS). All sulfated GAGs are usually attached to core proteins via covalent bonds, forming PGs, which are ubiquitously expressed in all mammals’ tissues, as integral components of the glycocalyx, ECM, intracellular granules and basement membranes. The remarkable structural diversity of PGs/GAGs enables them to interact with a variety of proteins, including those that are involved in the modulation of inflammation and lipid metabolism [18,23]. PGs/GAGs themselves can promote local immunity either directly by binding immune cell receptors such as integrins and Toll-like receptors (TLR), or indirectly by releasing immunoactive molecules such as cytokines and chemokines that are stored in the ECM structure [17,23,24,25]. GAGs are also involved in the subendothelial accumulation of atherogenic LDL, which is a critical event initiating in atherogenesis [25,26]. Lipoproteins, which diffuse from the plasma, are taken up and deposited in the intima by ionic interactions between negatively charged GAG sidechains of vascular proteoglycans and positively charged amino acid residues of apolipoprotein (apo) B100. The most representative proteoglycans in the artery wall are large chondroitin sulfate proteoglycan (CSPG), versican, small leucine-rich proteoglycans (SLRPs) containing chondroitin/dermatan sulfate chains, biglycan and decorin, and heparan sulfate proteoglycan (HSPG), perlecan. Among the proteoglycans mentioned, CSPGs play a crucial role in lipid retention, modification and final accumulation. It is well-known that binding of PGs/GAGs induces structural changes in LDLs, affecting both the apoB100 configuration and lipid composition. Hence, the long-term retention of lipoproteins in the vascular wall makes the LDLs more susceptible to oxidation and aggregation, which promotes foam cell formation and a proinflammatory response, in turn, further promoting the development of atherogenesis [26,27,28].

Due to their huge biological reactivity, GAGs play a significant role in initiating and controlling inflammatory events in a variety of pathological conditions, including RA. Several reports have shown that the physiological plasma/serum GAG profile is significantly altered in patients with RA, indicating impaired extracellular PGs/GAGs turnover, which is dependent on the control of disease activity [20,22]. For this reason, the main objective of this study was to determine the usefulness of the evaluation of particular types of plasma sulfated glycosaminoglycans (i.e., CS/DS and HS/H)—products of tissue proteoglycan degradation—as new biomarkers for monitoring the effectiveness of TNF-α inhibitors (TNFαI) in the treatment of RA. In addition, based on the hypothesis of a close relationship between extracellular matrix PGs/GAGs and the pathophysiology of accelerated atherosclerosis in RA, we also evaluated the effect of TNFαI on the vascular endothelial status by assessing the concentrations of selected biochemical markers of endothelial dysfunction such as sVCAM-1, MCP-1, MMP-9 and ADMA.

## 2. Materials and Methods

### 2.1. Patients and Samples

The study included 45 female patients above 18 years old who were diagnosed with RA based on the 2010 American College of Rheumatology (ACR)/European League Against Rheumatism (EULAR) criteria or the 1987 ACR criteria [29,30]. All patients had active RA, with a 28 joint disease activity score (DAS28) >5.1 at the baseline, despite treatment with at least two synthetic disease-modifying antirheumatic drugs (DMARDs) for a minimum of 6 months with each drug. The exclusion criteria were as follows: previous biological treatment, pregnancy/breastfeeding, other autoimmune diseases, acute or recent infection, cardiovascular disease, hypertension, diabetes or metabolic syndrome, thyroid disorders, renal or liver insufficiency, malignancies, smoking and alcohol consumption. In addition, none of the enrolled subjects received therapy known to have any effect on the cardiovascular system, including angiotensin-converting enzyme inhibitors, statins or hormone replacement therapy. Among the 45 RA patients, 22 received adalimumab (ADA; Humira) at 40 mg every other week as subcutaneous (SC) injection, 19 received etanercept (ETA; Enbrel) at 50 mg once weekly as SC injection and four received certolizumab pegol (CZP; Cimzia) 400 mg at 0, 2 and 4 weeks, and then 200 mg every 2 weeks as a SC injection over a 15-month period. Patients continued anti-rheumatic therapy, including methotrexate (MTX, 25 mg/week) and prednisone (≤7.5 mg/day) at a stable dose during the study. All patients were given folic acid in the dose of 5 mg/day. Presented in Table 1 are the variables of the demographic and clinical data for the RA patients eligible for anti-TNF-alpha therapy, as were obtained in our earlier investigations [31].

In addition, 20 age-matched healthy female volunteers from the Medical University of Silesia in Katowice, Poland were investigated as controls. Subjects were selected after obtaining their medical history and laboratory screening. Women with any history of cardiac disease and signs or symptoms from the heart, or with surgical procedures in the past 3 years, were excluded. All participants enrolled in the study had results of hematological and biochemical analyses within the reference range. None of the volunteers took steroidal or nonsteroidal anti-inflammatory drugs, smoked cigarettes or had any history of alcohol abuse. We chose women who could maintain a healthy weight and had a body mass index (BMI) <25 kg/m^2^.

Following an overnight fast, venous blood samples were collected in 3.8% sodium citrate (extraction and determination of plasma sulfated GAGs) and serum separator tubes (measurement of serum sVCAM-1, MCP-1, MMP-9 and ADMA). Serum was prepared by clotting the whole blood at room temperature for 30 min. Then, all samples were centrifuged at 1000× *g* for 10 min at 4 °C, and the serum and plasma supernatants were immediately frozen at −80 °C until the assessment time.

The study protocol was approved by the Ethical Committee of the Medical University of Silesia in Katowice (KNW/0022/KB/56/I/12/13). Written informed consent was obtained from all participants and the research was carried out in accordance with the conditions of the Declaration of Helsinki.

### 2.2. Clinical and Laboratory Analyses

The RA activity was evaluated at the baseline and after 3, 9 and 15 months of anti-TNF-α therapy using the DAS28 based on the erythrocyte sedimentation rate (DAS28-ESR). Patients who did not respond well to the treatment were excluded from the study. An adequate clinical response was defined—according to the Polish National Health Fund’s Drug Programs—as a reduction in DAS28-ESR by more than 1.2 after the first 3 months of biological therapy, and a further reduction in DAS28-ESR by 1.2 noted in subsequent medical examinations performed 9 and 15 months after the first dose of TNFαI.

Immunoglobulin M rheumatoid factor (IgM-RF, normal value ≤15 U/mL) and C-reactive protein (CRP, normal value <5 mg/L) were assessed using the Konelab Prime 30ISE (bioMérieux France, Craponne, France) biochemical analyzer. The anti-CCP antibodies (normal value ≤5 U/mL) were detected by an enzyme-linked immunosorbent assay (ELISA) from Euroimmun (Lubeck, Germany). In addition, ESR (normal range for women: 3–12 mm/h) was determined by the Westergren method (Sediplus^®^ S2000, Sarstedt, Germany).

Serum lipid parameters including total cholesterol (TC), high-density lipoprotein cholesterol (HDL-C), low-density lipoprotein cholesterol (LDL-C), triglycerides (TG) and lipoprotein (a) (Lp(a)) in all the studied subjects were assessed using a BioSystems A15 fully automatic biochemistry analyzer (BioSystems S.A., Barcelona, Spain). All lipid parameters were measured twice—at the baseline and after 15 months of anti-TNF-α therapy. Serum concentrations of total cholesterol, HDL-C, LDL-C and triglycerides were measured using enzymatic colorimetric methods (BioSystems S.A., Barcelona, Spain), whereas serum Lp(a) levels were detected by the latex immunoturbidimetry method (Pointe Scientific, Warsaw, Poland). The intra- and inter-assay coefficients of variation for all above biochemical parameters were less than 1.6% and 5.7%, respectively.

### 2.3. Isolation and Quantitative Analysis of Sulfated GAGs

The methods used for isolation and quantitative analysis of plasma sulfated GAGs were described previously in our earlier investigation [31]. The extraction and purification of sulfated GAGs were performed by ion exchange low-pressure liquid chromatography. The total amount of sulfated GAGs was quantified by a hexuronic assay according to the carbazole methods [31]. Then, isolated GAG samples (3 µg of hexuronic acid) were submitted to electrophoresis on cellulose acetate before and after treatment with specific bacterial lyases, i.e., chondroitinase ABC [EC 4.2.2.4] from *Proteus vulgaris* (Sigma-Aldrich, Steinheim, Germany) and a mixture of heparinases I [EC 4.2.2.7] and III [EC 4.2.2.8] from *Flavobacterium heparinum* (Sigma-Aldrich, Steinheim, Germany). Chondroitinase ABC was used to cleave CS/DS. Digestion was carried out in 0.05 M Tris HCl buffer, pH 8.0, containing 0.001 M sodium acetate and 0.02% (*w*/*v*) bovine serum albumin (BSA), for 24 h at 37 °C. In turn, depolymerization of GAGs with chondroitinase ABC in combination with heparinase I and III enabled CS/DS and HS/H to be eliminated, respectively. Digestion with heparinase I and III was conducted in 0.02 M Tris HCl buffer, pH 7.3, containing 0.05 M sodium chloride, 0.004 M calcium chloride and 0.01% (*w*/*v*) BSA for 24 h at 25 °C. Sample components that were resistant to the enzyme degradation were precipitated with 7 volumes of 99.8% ethanol for 24 h at 4 °C. After centrifugation (18,000× *g*, 30 min, 4 °C), the precipitate containing non-digested GAGs was dissolved in double-distilled water and subjected to electrophoresis resolution.

**Figure 1:**
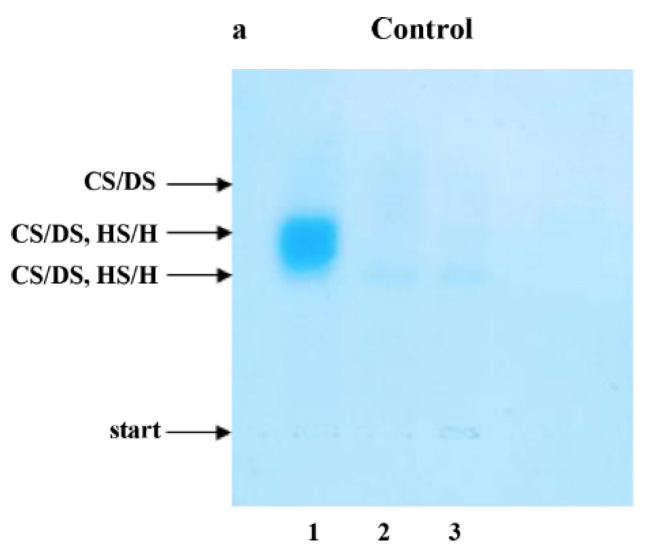

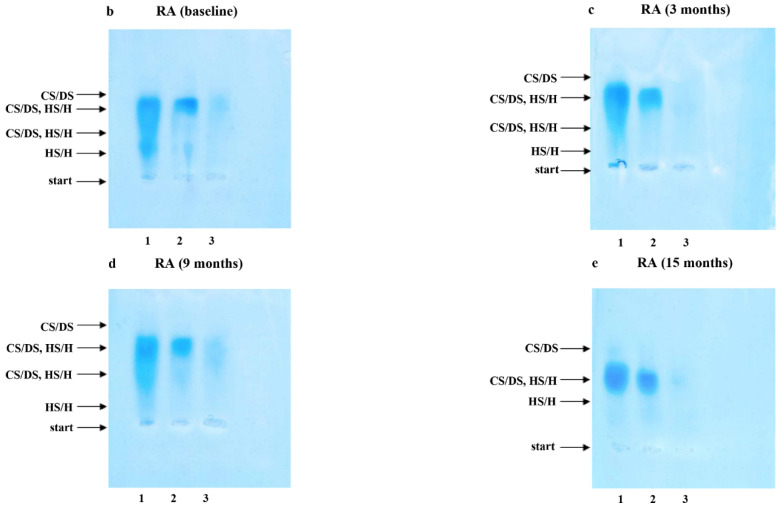
Typical electrophoretic patterns of plasma sulfated glycosaminoglycans (sGAGs) in healthy subjects (**a**) and patients with rheumatoid arthritis (RA) before (**b**) and after 3 (**c**), 9 (**d**) and 15 (**e**) months of anti-TNF-α therapy. GAGs were isolated from plasma samples and submitted to electrophoresis on cellulose acetate in 0.034 M Al_2_(SO_4_)_3_ before and after specific enzymatic degradation. Lane 1, untreated GAG sample (CS/DS, HS/H); lane 2, GAG sample resistant to the action of chondroitinase ABC (HS/H); lane 3, material resistant to the combined action of chondroitinase ABC and heparinase I and III. Comparison of electrophoretic patterns of intact material containing CS/DS and HS/H (line 1) with those obtained after chondroitinase ABC digestion (line 2) allowed to localize CS/DS in electrophoregrams (line 1–line 2 = CS/DS). Material resistant to chondroitinase ABC but susceptible to heparinase I and III digestion was identified as HS/H (line 2–line 3 = HS/H). CS/DS, chondroitin/dermatan sulfate; sGAGs, sulfated glycosaminoglycans; HS/H, heparan sulfate/heparin; RA, rheumatoid arthritis; anti-TNF-α, anti-tumor necrosis factor-α.

Electrophoresis on Cellogel cellulose acetate strips (Serva, Heidelberg, Germany) was carried out in 0.034 M aluminum sulfate buffer, at 5 V and 1 mA per 1 cm of strip width, for 2 h at 21 °C. The strips were stained with 0.15% (*w*/*v*) Alcian blue in 96% ethanol, re-distilled water and glacial acetic acid (10:14:1, *v*/*v*/*v*), according to Hronowski and Anastassiades [32], and were destained in the same solvent mixture but without Alcian blue. The identity of electrophoretic bands was confirmed by comparison of electrophoretic patterns of plasma GAG samples submitted to electrophoresis without any previous treatment (Figure 1a–e, lane 1) and depolymerized with agents specifically eliminating particular GAG types (Figure 1a–e, lanes 2–3). A quantitative analysis of the obtained electrophoregrams was performed using the gel documentation system G:BOX (Syngene, Cambridge, UK). Assessments were made at the baseline and 3, 9 and 15 months after TNFαI treatment.

### 2.4. Immunoassay of Vascular Endothelial Dysfunction Markers (sVCAM-1, MCP-1, MMP-9 and ADMA)

Serum levels of sVCAM-1 and MCP-1 were measured using commercially available ELISA kits from Diaclone SAS (Besancon Cedex, France). Levels of MMP-9 and ADMA were determined by ELISA kits from Cloud-Clone Corp. (Katy, TX, USA) and Immunodiagnostic AG (Bensheim, Germany), respectively. The analyses were performed according to the manufacturer’s instructions. The minimum detectable concentration was estimated to be = 0.6 ng/mL for sVCAM-1, <5.8 pg/mL for MCP-1, <0.055 ng/mL for MMP-9 and =0.29 μmol/L for ADMA. Testing of all samples in duplicate was completed in 1 day to eliminate the effects of inter-assay variation. The manufacturer’s intra-assay coefficients of variation (CVs) for the sVCAM-1 assay and ADMA assay ranged from 0.132% to 0.45% and from 5.8% to 7.9%, respectively. In addition, the CVs were = 1.8% for MCP-1 assay and <10% for MMP-9 assay.

### 2.5. Statistical Analysis

Statistical analysis of the obtained results was performed using the TIBCO Software, Inc., version 13.3; StatSoft Poland Sp. z o. o. 2022 (Palo Alto, CA, USA). Normality of the data distribution was evaluated by the Shapiro–Wilk test. Data non-normally distributed were log-transformed and re-checked for normality. Continuous variables are summarized as the mean ± SD if normally distributed, or as the median and interquartile range otherwise. Homogeneity of variance was evaluated using Levene’s test. Data were analyzed using RM-ANOVA (normal distributed data) with a sphericity check by means of Mauchly’s test, or using the RM-ANOVA Friedman’s test (non-normal data). Additional post-hoc analyses were carried out in the case of significant differences between subgroups based on the Tukey’s test (*p*-value <0.05) or the Mann–Whitney *U*-test (*p*-value obtained after application of Bonferroni correction, *p* <0.05/six possible comparisons). Moreover, the paired Student’s *t*-test (for normal distribution) or Wilcoxon’s rank sum test (for abnormal distribution) was used to compare the change in the same parameters in each RA patient before and after 15 months of anti-TNF-α treatment. *p*-values of less than 0.05 were considered significant. Spearman’s rank correlation coefficient was used to evaluate the relationships between circulating HS/H, indicators of disease activity and biomarkers of endothelial dysfunction (sVCAM-1, MCP-1, MMP-9 and ADMA) in female RA patients.

## 3. Results

### 3.1. Effectiveness of TNFαI Treatment

The demographic and clinical characteristics of RA patients during 15-month anti-TNF-α therapy (Table 1) were already described in our earlier investigations [31]. A total of 45 female RA patients met the eligibility criteria and were enrolled into the study, starting treatment with one of the SC-TNFαIs such as adalimumab, etanercept or certolizumab pegol. Out of a total of 45 female RA patients, 16 patients did not complete the study and were excluded from the analysis; the remaining 29 patients completed the 15 month follow-up period and are presented in this article. The primary reasons for study discontinuation of the 16 RA patients were as follows: lack of response (2 patients), loss of response (3 patients), therapy intolerance (3 patients), undergoing surgical procedures (4 patients) and withdrawal of consent to participate in therapy (4 patients). The biochemical, clinical and functional parameters of RA female patients who did not respond well to TNFαI treatment have been added in the Supplementary Material (Table S1). The final study population was 29 female RA patients who continued TNF-α therapy for 15 months, including 14 for ADA (48.27%), 13 for ETA (44.83%) and two for CZP (6.9%).

After initiation of TNFαI therapy, a significant improvement in clinical variables was observed in all RA patients. A significant decrease in disease activity was noted in the first assessment performed after 3 months of TNFαI therapy (DAS28-ESR 5.99 ± 0.50 at baseline vs. 4.00 ± 0.73, *p* <0.001; Table 1). Further reductions in the mean DAS28-ESR were noted in subsequent months. After 15 months of treatment, 79.31% and 20.69% of patients achieved clinical remission (defined as a DAS28 value ≤2.6) and low disease activity (defined as a DAS28 value <3.2), respectively. Furthermore, the rate of good response (DAS28-ESR improvement >1.2) according to EULAR criteria [33] at 3 months was 100% (29/29), and this effect persisted up to the 15th month. Regarding the inflammatory markers, significant reductions in CRP and ESR were observed in all patients following the anti-TNF-α treatment (Table 1).

### 3.2. Plasma Levels of Sulfated GAGs (CS/DS and HS/H)

The results of our study revealed that 15-month TNFαI therapy combined with MTX influence remodeling the plasma profile of the sulfated glycosaminoglycans in female RA patients. Typical electrophoretic patterns for plasma GAGs from healthy subjects and female RA patients before and after 3, 9 and 15 months of anti-TNF-α treatment are presented in Figure 1a–e. The electrophoretic analysis of plasma GAGs allowed us to identify: CS/DS and HS/H in plasma of all the investigated subject groups (Figure 1a–e). CS/DS were the major types of plasma GAGs in healthy subjects and RA patients both before and during the 15 months of TNFαI therapy (Table 2). CS/DSs derived from female RA patients were characterized by the increased structural heterogeneity and different mobility of these compounds compared to controls. Two (a fast and an intermediate) or three (a fast, an intermediate and a slow) CS/DS fractions of different electrophoretic mobilities were found in plasma of RA patients during TNFαI therapy, in contrast to three migration fractions in healthy subjects (Figure 1b–e vs. Figure 1a, lane 1). Interestingly, during the 15-month TNFαI treatment, the species with an intermediate electrophoretic mobility migrated less diffusely than in the baseline evaluation (Figure 1e vs. Figure 1b, lane 1). GAGs resistant to chondroitinase ABC digestion and susceptible to heparinase I and III action were identified as HS/H (Figure 1a–e, lanes 2 vs. 3). These types of GAGs also demonstrated electrophoretic heterogeneity. Three HS/H fractions of different mobilities were found in plasma of RA patients before and after 3 and 9 months of biological therapy, in contrast to two HS/H fractions in plasma of healthy subjects and RA patients who completed 15 months of treatment (Figure 1b–d vs. Figure 1a,e, lanes 2 vs. 3).

**Figure 2:**
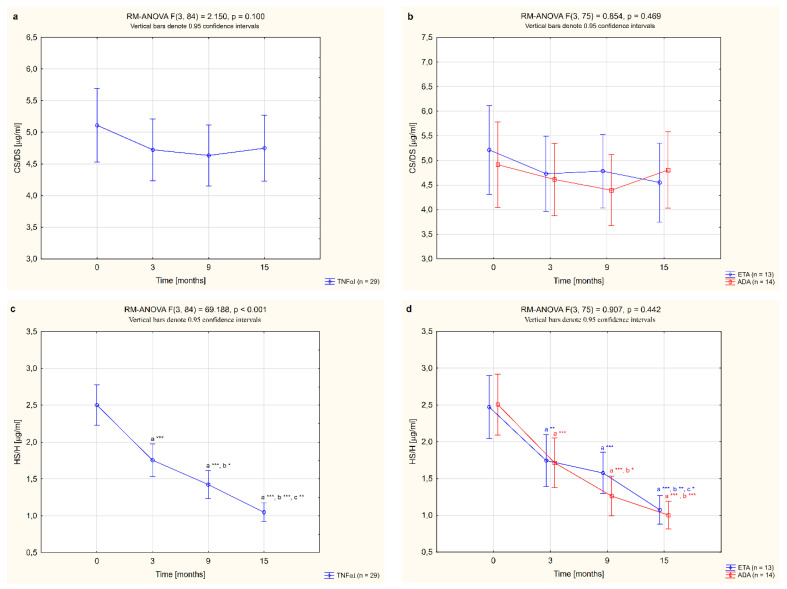
Changes to circulating levels of CS/DS (**a**,**b**) and HS/H (**c**,**d**) in all patients with rheumatoid arthritis (RA) during 15-month anti-TNF-α therapy and in groups divided by the type of treatment (ETA or ADA). ^a^ statistically significant differences compared to baseline; ^b^ statistically significant differences compared to 3 months after therapy; ^c^ statistically significant differences compared to 9 months after therapy. * *p* <0.05; ** *p* <0.01; *** *p* <0.001. ADA, adalimumab; CS/DS, chondroitin/dermatan sulfate; ETA, etanercept; HS/H, heparan sulfate/heparin; anti-TNF-α, anti-tumor necrosis factor-α.

Moreover, plasma CS/DS and HS/H levels were significantly higher in female RA patients before anti-TNF-α therapy in comparison to healthy subjects (*p* <0.01 and *p* <0.001, respectively; Table 2). CS/DS levels were not affected by the biological treatment (*p* = 0.100; Figure 2a) and did not differ significantly in patients after 15 months of TNFαI therapy compared to the control group (*p* = 0.094; Table 2). In the case of HS/H, three months after the initiation of anti-TNF-α therapy, a significant decrease in HS/H levels was observed in RA patients (*p* <0.001; Figure 2c). Continued TNFαI administration resulted in a further decline (*p* <0.001; Figure 2c), reaching HS/H levels—after 15 months of treatment—characteristic of the age-matched healthy controls (*p* = 0.414; Table 2).

**Table 2:** Circulating levels of chondroitin/dermatan sulfate (CS/DS) and heparan sulfate/heparin (HS/H) in healthy subjects and patients with rheumatoid arthritis (RA) before and after 15-month anti-TNF-α therapy.

Parameter	Healthy Subjects	RA Patients (*n* = 29)		*p*	
	A	T_0_ (Before TNFαI Therapy) B	T_3_ (15 Months After Starting TNFαI Therapy) C	A vs. B	A vs. C
CS/DS [µg/mL]	4.09 (3.72–4.42)	4.91 (3.91–6.45)	4.85 (3.75–5.89)	<0.01	0.094
HS/H [µg/mL]	0.93 (0.86–1.02)	2.35 (1.99–3.06)	1.03 (0.80–1.35)	<0.001	0.414

Data are presented as median or inter-quartile (25th–75th percentile) range. Data were analyzed using the Mann–Whitney *U*-test. CS/DS, chondroitin/dermatan sulfate; HS/H, heparan sulfate/heparin; RA, rheumatoid arthritis; TNFαI, tumor necrosis factor-α inhibitors.

In this study, we also assessed whether the type of TNF-α inhibitor used had an effect on the quantitative changes to the circulating sulfated GAGs. We compared alterations in plasma levels of CS/DS and HS/H in female RA patients who completed a 15-month anti-TNF-α therapy with ETA or ADA. No significant changes in plasma levels of CS/DS were observed following treatment with either ETA or ADA (*p* = 0.343 and *p* = 0.160, respectively; Figure 2b). On the other hand, administration of either ETA or ADA led to a significant decrease in plasma levels of HS/H (both *p* <0.001; Figure 2d). Meanwhile, there were no significant changes in the plasma levels of the analyzed ECM components, i.e., CS/DS and HS/H, depending on the type of TNF-α inhibitor used (*p* = 0.469 and *p* = 0.442, respectively; Figure 2b,d).

### 3.3. Serum Levels of Vascular Endothelial Dysfunction Markers (sVCAM-1, MCP-1, MMP-9 and ADMA)

**Figure 3:**
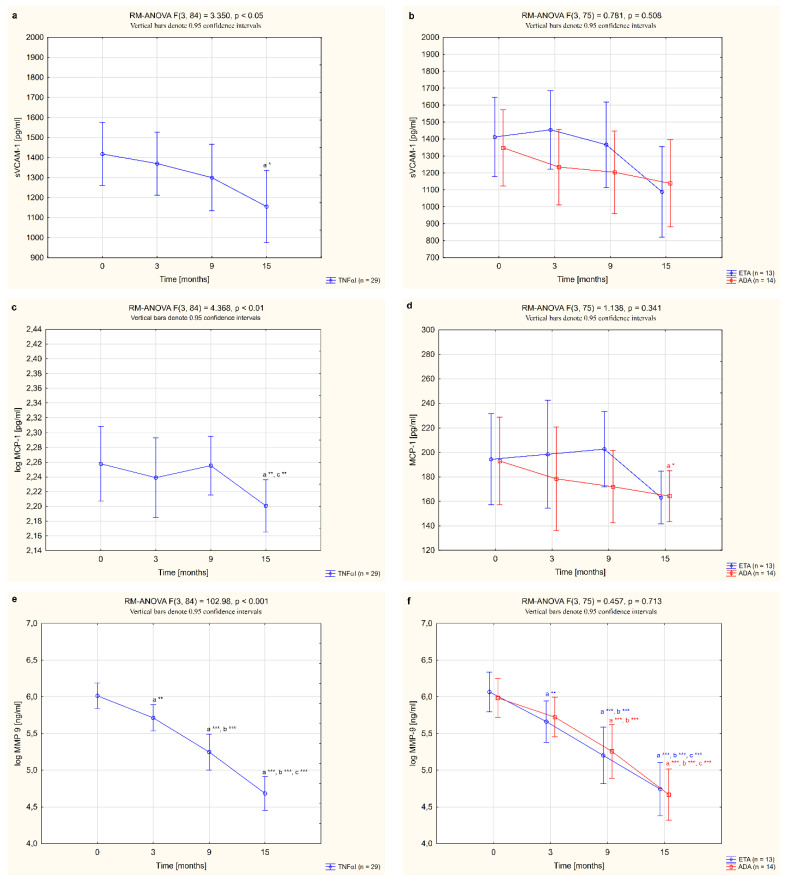

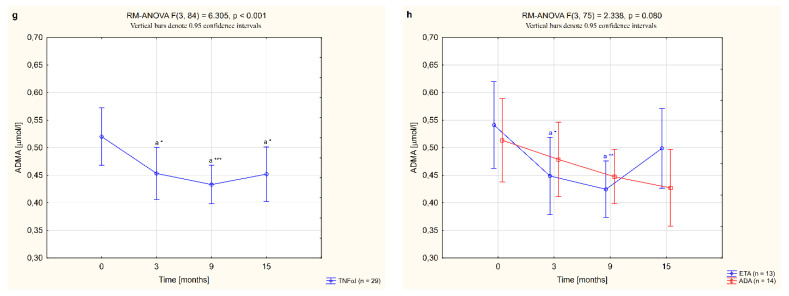
Changes to circulating levels of sVCAM-1 (**a**,**b**), MCP-1 (**c**,**d**), MMP-9 (**e**,**f**) and ADMA (**g**,**h**) in patients with rheumatoid arthritis during 15-month anti-TNF-α therapy and in groups divided by the type of treatment (ETA or ADA). ^a^ statistically significant differences compared to baseline; ^b^ statistically significant differences compared to 3 months after therapy; ^c^ statistically significant differences compared to 9 months after therapy. Data not normally distributed (MCP-1, MMP-9) were log-transformed before analyses. * *p* <0.05; ** *p* <0.01; *** *p* <0.001. ADA, adalimumab; ADMA, asymmetric dimethylarginine; ETA, etanercept; MCP-1, monocyte chemoattractant protein 1; MMP-9, matrix metalloproteinase 9; sVCAM-1, soluble of vascular cell adhesion molecule 1; TNF-α, tumor necrosis factor-α.

The serum concentrations of sVCAM-1, MCP-1, MMP-9 and ADMA in female RA patients undergoing biological therapy and in healthy subjects are presented in Table 3. Among the vascular endothelial dysfunction biomarkers analyzed, serum levels of sVCAM-1, MCP-1 and MMP-9 were higher in women with RA before anti-TNF-α therapy than in healthy individuals (*p* <0.05, *p* <0.05 and *p* <0.001, respectively; Table 3). There were no significant differences in ADMA levels between women with RA before TNFαI therapy and the control group (*p* = 0.106; Table 3). Treatment with anti-TNFα agents led to a significant reduction in the serum levels of all studied biomarkers, i.e., sVCAM-1, MCP1, MMP-9 and ADMA, compared to the baseline values. The serum concentrations of sVCAM-1 and MCP-1 in RA patients were especially diminished after 15 months of treatment with TNFαI (both *p* <0.05; Figure 3a,c) and were no longer different from the control values (*p* = 0.774 and *p* = 0.159, respectively; Table 3). Interestingly, serum levels of both MMP-9 and ADMA decreased significantly already after 3 months of anti-TNFα treatment (*p* <0.01 and *p* <0.05, respectively; Figure 3e,g). Continued TNFαI therapy prolonged and even increased the reduction in serum MMP-9 levels compared to the baseline evaluation (in all cases, *p* <0.001; Figure 3e) and compared to the values found in the third month (in all cases, *p* <0.001; Figure 3e). However, even at the end of the follow-up, levels of MMP-9 were still significantly higher in serum of RA patients than in healthy subjects (*p* <0.001; Table 3). Similarly, serum concentrations of ADMA in women with RA decreased with biological treatment (Figure 3g). The lowest serum levels of ADMA were noticed after 9 months of treatment (*p* <0.001; Figure 3g). Further TNFαI administration maintained ADMA suppression, although to a lesser extent than at 9 months of therapy (*p* <0.05; Figure 3g).

In our study, we also analyzed how the type of TNFαI affected changes in the serum levels of all biomarkers of vascular endothelial dysfunction in female RA patients. Overall, for women with RA who received ETA or ADA, neither TNF-α-blocking agent had an effect on circulating sVCAM-1 levels (*p* = 0.508; Figure 3b). We also demonstrated that serum levels of MCP-1 decreased significantly after 15 months of ADA treatment (*p* <0.05; Figure 3d), but were not significantly changed by treatment with ETA (*p* = 0.143; Figure 3d).

Nonetheless, 3 and 9 months of therapy with etanercept significantly suppressed serum MMP-9 (both *p* <0.001; Figure 3f) and ADMA concentrations (*p* <0.05 and *p* <0.001, respectively; Figure 3h). Interestingly, suppression of MMP-9 was continued to the end of the study. Similarly, administration of ADA led to a significant decrease in serum levels of MMP-9 in RA patients after 9 and 15 months of therapy (both *p* <0.001; Figure 3f). The serum levels of ADMA tended to reduce in RA patients after 15 months of ADA therapy (*p* = 0.050; Figure 3h). In general, there were no significant differences in the serum levels of the assessed endothelial inflammation markers, i.e., sVCAM-1, MCP-1, MMP-9 and ADMA (*p* = 0.508, 0.341, 0.713 and 0.080, respectively; Figure 3b,d,f,h), depending on the type of TNF-α inhibitor used.

### 3.4. Analysis of the Relationships between Circulating Heparan Sulfate/Heparin, Endothelial Dysfunction Markers and Clinical and Laboratory Indicators of Disease Activity

The analysis of the relationship between circulating HS/H and selected endothelial dysfunction markers (sVACAM-1, MCP-1, MMP-9 and ADMA), as well as clinical (DAS 28-ESR, SW, TEN, VAS) and laboratory (ESR, CRP) indicators of disease activity in RA patients, at the beginning and after 15 months of anti-TNF-α therapy, are presented in Table 4. Before the first dose of TNFαI, a significant positive correlation was detected between the circulating HS/H and levels of serum vascular endothelial dysfunction markers, such as MCP-1 (r = 0.398; *p* <0.05) and ADMA (r = 0.396; *p* <0.05), respectively (Table 4). Furthermore, plasma HS/H levels significantly correlated with DAS28-ESR (r = 0.408; *p* <0.05) and TEN (r = 0.453; *p* <0.05), respectively (Table 4). During TNFαI treatment, these correlations were less or not significant (data not shown). No correlations between HS/H levels, the ESR, CRP, SW and VAS of the patients and the serum concentrations of sVCAM-1 or MMP-9 were noted (Table 4).

## 4. Discussion

Mortality in patients with RA is quite high, which is largely due to cardiovascular events not fully explained by other classic cardiovascular risk factors. The exact mechanisms for the development of premature atherosclerosis during RA are still unclear, but persistent inflammation has been postulated to promote endothelial dysfunction and vascular injury. In addition to that, increased local and systemic expression of specific inflammatory mediators in RA progression is closely associated with extensive ECM remodeling and abnormal release of PGs/GAGs into the circulation [18,22]. We previously showed that disturbances affecting the ECM during RA were reflected in quantitative changes to the circulating GAGs, i.e., peptide-bound and free-GAG chains, which represent components of intact PGs, mainly of hepatic and endothelial origin, as well as products of tissue PG breakdown. Total levels of plasma GAGs in RA patients were higher in comparison to healthy subjects [31]. In the present investigation, we demonstrated that an increase in the circulating GAGs in female patients with RA before TNFαI treatment resulted mainly from elevated CS/DS and HS/H contents. Increased structural diversity of sulfated types of GAGs was also observed. The accuracy of this observation is supported by recent studies [20,34]. Furthermore, a significant remodeling of the circulating GAG profile has also been evidenced in various inflammatory diseases, such as acute pancreatitis, carotid atherosclerosis and sepsis [35,36,37]. We also observed a TNF-α blockade affect manifested by a rapid reduction in the levels of HS/H, as well as endothelial dysfunction markers, following administration of TNFαI. These changes may explain the beneficial effect observed by anti-TNF-α therapy on the mechanisms associated with accelerated atherosclerosis in patients with RA.

The blood accumulation of sulfated GAGs in the course of RA may support a role of these macromolecules in inflammatory states. Among sulfated GAGs, the HS/H family is the best-studied in terms of the biointeractions that affect cell behavior via the striking plethora of the specific HS/H cell ligands [18,23]. Indeed, HS/H chains, due to their great structural heterogeneity, can bind and interact with a wide variety of proteins, such as L-selectin, leukocyte integrin Mac-1 (CD11b/CD1), chemokine ligand (CXCL) 8, CXCL1 and MCP-1/CC chemokine ligand 2 (CCL2), which modulate recruitment and infiltration of inflammatory cells, particularly lymphocytes and neutrophils from the vasculature, to the site of inflammation [24]. Interestingly, it is not only the heparan sulfate chains attached to the endothelial glycocalyx HSPGs that are capable of inflammatory signaling; soluble HS also modulates the binding of chemokines or selectins to their receptors [24]. The increased levels of plasma HS/H in female RA patients before TNFαI treatment may be attributed to the heparanase (HPSE) effect. Heparanase is an endo-β-D-glucuronidase and the only known mammalian enzyme capable of cleaving heparan sulfate chains both on the cell surface and in the ECM, thereby regulating the release of HS into the circulation [24,38,39]. It was demonstrated that heparanase-induced shedding of syndecan 1 and HS/H chains represents an important mechanism of chronic inflammation, leading to loss of glycocalyx integrity and subsequent vascular dysfunction [20,39]. Elevated heparanase expression has been shown in several inflammatory pathologies, such as cardiovascular disease, diabetes, kidney disease and sepsis [24,40,41]. High activity of heparanase was also found in synovial fluid and the synovium of RA patients [42]. Not surprisingly, the major proinflammatory cytokines involved in RA pathogenesis, TNF-α and IL-1, have been shown to upregulate heparanase expression in endothelial cells [43,44], which may be a potential cause of an increased risk of atherosclerosis associated with this arthropathy. HPSE degrades HS in regions of high sulfation, resulting in the release of 5–7 kDa soluble HS fragments, which induce inflammatory signaling and cytokine upregulation through a direct interaction with TLR4 and possibly TLR2, and tyrosine kinase receptor EphA3 [39,41,45]. These findings are consistent with the results of Jura-Półtorak et al. [20], who revealed a close relationship between increased plasma levels of HS/H in RA patients and DAS28, as well as inflammatory markers, such as CRP and ESR. Plus, in our study, before the initial TNFαI administration, plasma concentrations of HS/H in women with RA were positively correlated with the increase in DAS28. Taken together, these observations indicate a widespread role of heparanase and HS/H in the pathophysiology of the vessel wall during several athero-prone conditions, including RA.

Since HSPGs have been proposed to be antiatherogenic [46], we can suggest that elevated circulating HS/H levels, mainly due to increased arterial HSPG degradation, may be an important indicator of endothelial dysfunction in RA patients. Reduced levels of HSPGs/HS were detected in fully developed atherosclerotic lesions derived from non-diabetic and type 2 diabetes mellitus patients, and from those with carotid stenosis, respectively [47,48]. Therefore, it is plausible that impaired distribution of arterial HSPGs/HS is driving the initiation and progression of atherosclerosis. In line with this suggestion, we also found a positive association between circulating HS/H and levels of vascular endothelial dysfunction biomarkers, such as MCP-1 and ADMA. Loss of arterial HSPGs has been shown to increase LDL binding to the endothelial matrix in vitro, indicating that HS may interfere with lipoprotein retention [26,46,49]. HS also reduces vascular wall permeability to LDLs and decreases monocyte binding. In addition, release of HS fragments carrying growth factors such as vascular endothelial growth factor (VEGF) or fibroblast growth factor 2 (FGF2) may induce angiogenesis and smooth muscle cell migration and proliferation [26,27,48,49,50,51]. However, perlecan has also been shown to be a pro-atherogenic HSPG in mouse models of atherosclerosis [27,46].

Similar to the plasma HS/H, it is believed that the circulating CS/DS can play an important role in the inflammation-related diseases. It is well-known that the major human GAG in plasma is undersulfated CS, which circulates while covalently attached to the proteoglycan bikunin, circulates as a main product of tissue catabolism or is secreted by activated mononuclear leukocytes [36,52,53]. Bikunin is principally synthesized in hepatocytes and occurs in blood as the light chain of inter-α-trypsin inhibitor (ITI) family molecules, i.e., pre-α-inhibitor (PαI) and inter-α-inhibitor (IαI) [36,54]. Although the physiological function of bikunin still remains to be revealed, several reports suggest that it exhibits anti-inflammatory properties that may contribute to termination of the inflammatory process [36,54,55]. Besides its role as a serine protease inhibitor, ITI family members are implicated in downregulating the production of proinflammatory cytokines and attenuating the complementary activation, as well as suppressing neutrophil accumulation and activation [54]. Under inflammatory conditions, plasma IαI components are strongly modified, which is associated with their high susceptibility to proteolysis by numerous proteinases involved in inflammation—namely plasmin, thrombin, kallikrein and neutrophil elastase. Moreover, it has been documented that hepatic synthesis of IαI components is downregulated, whereas that of PαI is upregulated. Indeed, low plasma concentrations of IαI have been reported in patients with septic shock, rheumatoid arthritis and acute bacterial infection [54,55,56]. Thus, a deficiency of CS-containing IαI glycoproteins may adversely affect the course of chronic inflammatory diseases, including RA.

Moreover, two closely related essential components of endothelial glycocalyx and articular cartilage matrix, i.e., soluble biglycan (sBGN) and soluble decorin (sDCN), are other molecules known to influence the pool of the circulating CS/DS in RA patients. In fact, several studies showed that sBGN and sDCN were present in synovial fluid of patients with advanced osteoarthritis and RA [57]. Furthermore, elevated levels of soluble biglycan were detected in the serum of mice subjected to collagen-induced RA [58]. Interestingly, similar to fragments of the tissue HS chains, soluble SLRPs released into the body fluids as a result of tissue injury could potentially function as an endogenous danger signal. By binding to TLR2/TLR4 in some cell types, i.e., macrophages or chondrocytes, soluble biglycan can induce and perpetuate a pro-inflammatory response through the synthesis of various inflammatory mediators, such as TNF-α, IL-1β, IL-6 and IL-17, as well as matrix metalloproteinases such as MMP-1, MMP-9 and MMP-13, which promote further ECM degradation [59,60,61]. It has also been found that sBGN induces the expression of ICAM-1 and MCP-1 in human aortic valve interstitial cells via TLR-2/4 and the extracellular signal-regulated kinase-1/2 (ERK-1/2) pathway [62]. This evidence indicates that the circulating degradation products of tissue PGs/GAGs are biologically active participants in perpetuating inflammation, suggesting an important role in the development of premature atherosclerosis during RA.

As such, PGs/GAGs play a crucial role in both the development and suppression of inflammation; it seems that circulating sulfated GAGs may be an interesting diagnostic and therapeutic target in RA. Indeed, in this study, we showed, for the first time to our knowledge, that the effective long-term treatment of RA with TNFαI in combination with MTX significantly lowered plasma HS/H levels. These findings suggest that anti-TNF-α treatment aimed at suppressing the inflammatory response might also positively affect the metabolism of cell-surface and extracellular matrix HSPGs/HS. In addition, this significant decrease in the levels of plasma HS/H indicates that circulating HS/H seem to be useful as biomarkers for monitoring TNF-α inhibitors’ effectiveness, as well as disease activity, in patients with RA undergoing the anti-TNF-α therapy. Our observations are in good agreement with the findings of Jura-Półtorak et al. [20], who showed that HS/H plasma levels in RA patients were dependent on the disease activity.

Considering our results, we can propose that decreased levels of HS/H during 15 months of anti-TNF-α therapy might be due to reduced enzymatic and nonenzymatic degradation of both cell-associated and matrix HSPGs/HS. Under inflammatory conditions, several factors, such as heparanases, various MMPs (i.e., MMP-1, MMP-2, MMP-7 and MMP-9) and reactive oxygen/nitrogen species (ROS/RNS), have been shown to mediate HSPGs/HS degradation [21,39,44,63,64]. Cysteine proteases, such as cathepsins B and L, are involved in glycocalyx shedding and extracellular matrix remodeling [21,63]. With regard to the enzymes discussed earlier, little is known about the effect of TNFαI treatment on both HPSE and cathepsin activity in RA patients. In contrast, the levels of various MMPs in serum of RA patients were found to be downregulated by anti-TNFα therapy [65,66,67]. In the present study, we observed a reduction in serum MMP-9 levels in women with RA after 3 months of anti-TNFα treatment. Moreover, we demonstrated that prolonged TNFαI administration sustained suppression of plasma MMP-9 levels. Plus, in agreement with our results, Kotani et al. [66] and Klimiuk et al. [67] reported significantly lower MMP-9 levels in RA patients treated with infliximab. It has also been demonstrated in human blood monocytes/macrophages in vitro that etanercept significantly reduces MMP-9 gene expression [68]. For that reason, serum MMP-9 levels in patients with coronary artery disease are high and correlated with the incidence of cardiovascular events [69,70]. Accordingly, we can propose that the reduction in serum MMP-9 levels by anti-TNF-α treatment may help inhibit the shedding of cell surface HSPGs, thereby preventing progression of atherosclerotic lesions in RA patients.

Another mechanism of alterations to the plasma HS/H level in RA patients may be connected to the ability of TNFαI to reduce oxidative stress, which is considered the key driver of accelerated atherosclerosis in RA. It is well-known that ROS and RNS can cleave the glycocalyx, both directly through GAG fragmentation and indirectly through MMP activation [63,64]. Indeed, TNF-α blocking therapy has been shown to reduce circulating levels of oxidative stress markers in RA patients [71,72]. Moreover, Cacciapaglia et al. [72] reported that treatment with TNFαI could suppress ROS generation in RA patients by reducing systemic inflammation. Thus, decreased levels of circulating HS/H, along with significantly decreased disease activity markers, such as DAS28, CRP and ESR, under anti-TNF-α treatment indicate that only good control of disease activity in RA can improve HSPGs/HS metabolism and delay cardiovascular events.

In contrast to HS/H, plasma levels of CS/DS were not affected by the anti-TNF-α treatment. To our knowledge, the effect of TNFαI in combination with MTX on the circulating sulfated GAGs has not been evaluated so far. To date, only one study showed changes in plasma CS/DS in RA patients treated with DMARDs (i.e., MTX or sulfosalasine) with respect to disease activity [20]. Contrary to our results, it was shown that plasma CS levels decreased with disease activity [20]. The explanation for these contrasting results may lie in the different methods used to determine sulfated GAGs and the heterogeneity of the populations evaluated, as well as the types of anti-rheumatic drugs used. Studies with larger number of patients may be necessary to further explore the impact of TNFαI on CS/DS metabolism in RA.

Finally, in the present study, we studied the effect of TNFαI on vascular endothelial dysfunction markers in female RA patients. It is well-established that various chemokines, along with adhesion molecules, promote the migration and penetration of inflammatory cells into the sub-endothelial space and synovium, leading to the initiation and progression of RA [9,16]. Elevated levels of soluble cell adhesion molecules such as sVCAM-1, and chemokines such as MCP-1, have been reported during endothelial dysfunction, atherosclerosis and chronic inflammatory diseases, including RA [8,73,74,75,76,77,78,79,80]. Similarly, in our study, both serum sVCAM-1 and MCP-1 levels were higher in female RA patients before the initial injection of TNFαI in comparison to the healthy individuals. Furthermore, effective 15-month anti-inflammatory treatment with TNFαI led to a significant reduction in sVCAM-1 and MCP-1 levels to the values observed in the controls. We also found that adalimumab therapy could be associated with a better normalization of MPC-1 than etanercept therapy. These results are in line with previous studies by Klimiuk et al. [75,76,77] and Kageyama et al. [78]. Klimiuk et al. [75,76] demonstrated that long-term anti-TNFα therapy with etanercept or infliximab significantly reduced serum sVCAM-1 levels in patients with RA. Kageyama et al. [78] reported significantly lower serum MCP-1 levels in RA patients at 3 and 6 months after the initial treatment with etanercept. Plus, in agreement with our results, Klimiuk et al. [77] confirmed that circulating levels of MCP-1 were significantly downregulated following 12-month therapy with etanercept. Moreover, these researchers [77,78] found a significant correlation between serum MCP-1 levels and DAS28 or ESR, indicating a role for MCP-1 in RA-associated inflammation. On the contrary, Fabre et al. [81] showed that 3-month treatment with etanercept did not change serum MCP-1 levels in RA patients responsive to anti-TNF-α treatment. Furthermore, another recent study [82] in patients with RA reported an increase in serum MCP-1 levels after 12 months of anti-TNF-α treatment. These discrepancies are not easily explained, but differences in the race, gender and age of the patients, and their BMI, exercise and disease duration, as well as CVD risk factors, may play a significant role. In addition, it has been reported that genetic variations could be a key factor in the variability of plasma levels of MCP-1 [83].

Regarding serum ADMA, we found a rapid, significant decrease in its levels after 3 months of TNFαI treatment; however, baseline ADMA values were comparable to healthy subjects. This reduction persisted throughout the study, and the lowest serum levels of ADMA were noted after 9 months of anti-TNF-α therapy. Moreover, our data indicated that etanercept therapy appeared to have a better effect on decreasing ADMA levels than adalimumab therapy. ADMA as a competitive inhibitor of nitric oxide synthases (NOSs) involved in the development of endothelial dysfunction [14]. Growing evidence has revealed that circulating levels of ADMA are associated with cardiovascular events, including coronary heart disease and acute stroke [84]. In RA, serum/plasma ADMA levels have been shown to be increased in female RA patients compared to healthy controls [8,85,86,87,88,89]. However, in some studies, circulating levels of ADMA in RA patients have been reported as normal [90,91]. According to our results, Spinelli et al. [87] described a reduction in serum ADMA levels after 3 months of anti-TNF-α treatment with etanercept. Similar results were also found in the serum of patients with early-stage RA after 12 months of treatment with adalimumab or synthetic DMARDs [88]. However, other investigators did not find any effect of 18-month conventional or biological DMARD therapy [89]. Finally, Sando et al. [92] reported no change in serum ADMA levels after 2-week and 3-month treatment of RA patients with adalimumab, etanercept or infliximab. Such large discrepancies in ADMA levels, both before and after TNFI treatment, found by various researchers, may be related, among others, to the impact of various factors on the plasma levels of this marker. According to the results of the meta-analysis by Zafari et al. [93], RA patients >50 years of age had significantly higher ADMA levels than healthy subjects of the same age. What’s more, methodological differences (enzymatic vs. chromatographic assays), the diversity of the study design and the heterogeneity of the assessed populations, as well as the limited number of patients in both ours and some other studies, could influence the results.

In summary, decreased serum levels of sVCAM-1, MCP-1 and ADMA after successful anti-TNF-α therapy may indicate reduced endothelial activation/dysfunction, mainly by achieving inflammation control, which is an important mechanism for preventing the development of cardiovascular events in RA patients.

## 5. Conclusions

To our knowledge, this study is the first report showing decreased levels of circulating HS/H in women with RA following effective anti-TNF-α therapy. These changes were related to the beneficial effect of TNFαI on the levels of circulating biomarkers of vascular endothelial dysfunction (i.e., sVACAM-1, MCP-1, MMP-9 and ADMA), which may be clinically relevant and contribute to the reported protective effect of anti-TNF-α therapy against cardiovascular risk in RA patients.

The normalization of plasma levels of HS/H in women with RA suggests that early, aggressive treatment of RA aimed at suppressing the inflammatory response contributes to an improvement in the balance between the synthesis, secretion, modification and degradation of HSPG/HS at the cell surface, thereby reducing endothelial dysfunction. Thus, quantitative changes in circulating HS/H during biological therapy may be useful for monitoring anti-TNF-α treatment in RA patients. However, due to the relatively small number of patients in the study group, further studies are needed to confirm our results.

## Figures and Tables

**Table 1:** Alterations to biochemical, clinical and functional measures during 15-month anti-TNF-α therapy.

Parameter	RA Patients (*n* = 29)			
	Before TNFαI Therapy		After Starting TNFαI Therapy	
	T_0_	T_1_ (3 Months)	T_2_ (9 Months)	T_3_ (15 Months)
Age [years], mean (SD)	44.38 (14.17)			
Disease duration [years], median (IQR)	5 (3–8)			
BMI [kg/m^2^], mean (SD)	21.25 (2.28)			
RF positive, n (%)	29 (100)			
Anti-CCP positive, n (%)	29 (100)			
SW, n median (IQR)	6 (5–10)	3 (2–3) ^a^	0 (0–1) ^a,b^	0 (0–0) ^a,b,^
TEN, n median (IQR)	14 (10–20)	5 (3–7) ^a^	2 (1–2) ^a,b^	0 (0–1) ^a,b,c^
VAS, [0–100 mm] median (IQR)	80 (80–80)	50 (35–55) ^a^	25 (10–30) ^a,b^	10 (5–20) ^a,b,c^
DAS28-ESR, mean (SD)	5.99 (0.50)	4.00 (0.73) ^a^	2.74 (0.72) ^a,b^	2.06 (0.64) ^a,b,c^
Disease activity, *n* (%)				
High (>5.1)	29 (100)	2 (6.90)	0	0
Moderate (>3.2 and ≤5.1)	0	24 (82.76)	6 (20.69)	0
Low (≤3.2 and >2.6)	0	3 (10.34)	12 (41.38)	6 (20.69)
Remission (≤2.6)	0	0	11 (37.93)	23 (79.31)
ESR [mm/h], median (IQR)	15.0 (10.0–31.0)	10.0 (8.0–17.0)	10.0 (8.0–14.0) ^a^	11.0 (8.0–14.0) ^a^
CRP [mg/l], median (IQR)	5.0 (4.0–9.2)	4.0 (2.0–4.0)	3.0 (1.30–4.0) ^a^	2.0 (1.0–4.0) ^a^
TC [mg/dl], mean (SD)	226.69 (23.73)			265.24 (64.51) ^a^
HDL-C [mg/dl], median (IQR)	57.9 (39.9–88.7)			79.7 (44.7–93.2) ^a^
LDL-C [mg/dl], mean (SD)	70.47 (23.73)			98.52 (27.98) ^a^
TG [mg/dl], median (IQR)	116.1 (91.9–138.9)			142.3 (126.3–154.6)
Non-HDL-C [mg/dl], median (IQR)	183.7 (100.3–208.0)			196.9 (147.8–238.1) ^a^
Lp(a) [mg/dl], median (IQR)	13.80 (8.80–32.20)			11.00 (9.20–33.10)

Data are presented as mean ± standard deviation (SD), median, inter-quartile (IQR, 25th–75th percentile) range or percentage (%). Anti-CCP, anti-cyclic citrullinated peptide antibody; BMI, body mass index; CRP, C-reactive protein; DAS28-ESR, 28 joint disease activity score based on erythrocyte sedimentation rate; ESR, erythrocyte sedimentation rate; HDL-C, high-density lipoprotein cholesterol; LDL-C, low-density lipoprotein cholesterol; Lp(a), lipoprotein (a); non-HDL-C, non-high-density lipoprotein cholesterol; RA, rheumatoid arthritis; RF, rheumatoid factor; SW, swollen joint count; TEN, tender joint count; TC, total cholesterol; TG, triglyceride; TNFαI, tumor necrosis factor-α inhibitors; VAS, visual analog scale. Differences noted for all variables (except for data related to DAS28-ESR and outcomes such as serum levels of TC, LDL-C, TG and non HDL-C) were considered significant at *p* <0.008333 by applying a Bonferroni correction. ^a^ statistically significant differences compared to T_0_; ^b^ statistically significant differences compared to T_1_; ^c^ statistically significant differences compared to T_2_.

**Table 3:** Circulating levels of sVCAM-1, MCP-1, MMP-9 and ADMA in healthy subjects and patients with rheumatoid arthritis (RA) before and after 15-month anti-TNF-α therapy.

Parameter	Healthy Subjects A	RA Patients (*n* = 29)		*p*	
		T_0_ (Before TNFαI Therapy) B	T_3_ (15 Months After Starting TNFαI Therapy) C	A vs. B	A vs. C
sVCAM-1 [pg/mL]	1117.09 (423.39)	1417.65 (414.19)	1155.20 (473.39)	<0.050	0.774
MCP-1 [pg/mL]	141.25 (118.09–163.41)	174.58 (142.40–223.28)	152.12 (137.53–176.10)	<0.050	0.159
MMP-9 [ng/mL]	54.93 (26.39–74.74)	377.82 (264.64–656.64)	97.80 (70.16–164.24)	<0.001	<0.001
ADMA [µmol/l]	0.46 (0.35–0.52)	0.52 (0.44–0.56)	0.44 (0.35–0.52)	0.106	0.912

Data are presented as mean ± standard deviation (SD), median or inter-quartile (25th–75th percentile) range. Data analyzed using Student’s *t*-test or the Mann–Whitney *U*-test. ADMA, asymmetric dimethylarginine; MCP-1, monocyte chemoattractant protein 1; MMP-9, matrix metalloproteinase 9; RA, rheumatoid arthritis; sVCAM-1, soluble of vascular cell adhesion molecule 1; TNFαI, tumor necrosis factor-α inhibitors.

**Table 4:** Relationship between circulating levels of heparan sulfate/heparin (HS/H) and clinical and laboratory indicators of disease activity, as well as markers of endothelial dysfunction (sVCAM-1, MCP-1, MMP-9 and ADMA), in patients with rheumatoid arthritis (RA) before and after 15-month anti-TNF-α therapy.

Parameter	RA Patients (*n* = 29)			
	T_0_ (Before TNFαI Therapy)		T_3_ (15 Months After Starting TNFαI Therapy)	
	HS/H [µg/mL]		HS/H [µg/mL]	
CRP [mg/l]	−0.356	*p* = 0.063	0.151	*p* = 0.444
ESR [mm/h]	−0.109	*p* = 0.582	−0.022	*p* = 0.911
SW, *n*	0.123	*p* = 0.533	−0.006	*p* = 0.975
TEN, *n*	0.453	*p* <0.05	−0.077	*p* = 0.697
VAS [0–100 mm]	0.117	*p* = 0.554	0.356	*p* = 0.063
DAS28-ESR	0.408	*p* <0.05	−0.059	*p* = 0.766
sVCAM-1 [pg/mL]	0.111	*p* = 0.574	−0.142	*p* = 0.470
MCP-1 [pg/mL]	0.398	*p* <0.05	0.083	*p* = 0.674
MMP-9 [ng/mL]	0.152	*p* = 0.441	0.308	*p* = 0.111
ADMA [µmol/L]	0.396	*p* <0.05	0.114	*p* = 0.563

Data expressed as r values (correlation coefficient) according to Spearman’s rank correlation. Correlations were considered significant at *p* <0.05. ADMA, asymmetric dimethylarginine; CRP, C-reactive protein; DAS28-ESR, 28 joint disease activity score based on erythrocyte sedimentation rate; ESR, erythrocyte sedimentation rate; HS/H, heparan sulfate/heparin; MCP-1, monocyte chemoattractant protein 1; MMP-9, matrix metalloproteinase 9; RA, rheumatoid arthritis; sVCAM-1, soluble of vascular cell adhesion molecule 1; SW, swollen joint count; TEN, tender joint count; TNFαI, tumor necrosis factor-α inhibitors; VAS, visual analog scale.

## Data Availability

Data are contained within the article.

## Supplementary Materials

The following supporting information can be downloaded at: https://www.mdpi.com/article/10.3390/jcm11144213/s1, Table S1. Changes in biochemical, clinical and functional markers in women with RA without responding to or loss of response to TNFαI therapy over time.

## Abbreviations

ADA, adalimumab; ADMA, asymmetric dimethylarginine; BMI, body mass index; CRP, C-reactive protein; CS, chondroitin sulfate; CSPG, chondroitin sulfate proteoglycan; CVD, cardiovascular disease; CZP, certolizumab pegol; DAS28-ESR, 28 joint disease activity score based on erythrocyte sedimentation rate; DMARD, disease-modifying antirheumatic drug; DS, dermatan sulfate; EAM, extra-articular manifestations; EC, endothelial cell; ECM, extracellular matrix; eNOS, endothelial nitric oxide synthase; ESR, erythrocyte sedimentation rate; ETA, etanercept; H, heparin; HPSE, heparanases; HS, heparan sulfate; HSPG, heparan sulfate proteoglycan; IαI, inter-α-inhibitor; ICAM-1, intercellular adhesion molecule; IL, interleukin; ITI, inter-α-trypsin inhibitor; KS, keratan sulfate; LDL, low-density lipoproteins; MCP-1, monocyte chemoattractant protein-1; MI, myocardial infarction; MMP, matrix metalloproteinase; MTX, methotrexate; NO, nitric oxide; NOS, nitric oxide synthase; PαI, pre-α-inhibitor; PG, proteoglycan; RA, rheumatoid arthritis; RNS, reactive nitrogen species; ROS; reactive oxygen species; sBGN, soluble biglycan; SC, subcutaneous; sDCN, soluble decorin; sGAG, sulfated glycosaminoglycan; SLRP, small leucine-rich proteoglycan; sVCAM-1, soluble vascular cell adhesion molecule-1; SW, swollen joint count; TEN, tender joint count; TLR, Toll-like receptors; TNF-α, tumor necrosis factor-alpha; TNFαI, tumor necrosis factor-α inhibitors; VAS, visual analog scale.
