# Supplementary material for: TNF-α Inhibitors in Combination with MTX Reduce Circulating Levels of Heparan Sulfate/Heparin and Endothelial Dysfunction Biomarkers (sVCAM-1, MCP-1, MMP-9 and ADMA) in Women with Rheumatoid Arthritis
Source: J Clin Med. 2022 Jul 20;11(14):4213. doi: 10.3390/jcm11144213 (PMC9320287; doi:10.3390/jcm11144213)
Supplement: Supplementary file 1 [file jcm-11-04213-s001.zip › jcm-1782425-supplementary.pdf]

Table S1. Changes in biochemical, clinical and functional markers in women with RA without responding to or loss of response to TNFαI therapy over time.

No Of Patients	Parameter	RA Patients With Primary Fail To Respond To TNFαI Therapy (n = 2)			
		Before TNFαI Therapy	After Starting TNFαI Therapy		
		T_0	T_1 (3 Months)	T_2 (9 Months)	T_3 (15 Months)
**Patient 1**	Age [years]	45			
	Disease duration [years]	15			
	SW, n	12	10	-	-
	TEN, n	14	28	-	-
	VAS, [0-100 mm]	80	80	-	-
	DAS28-ESR	6.36	7.40	-	-
	ESR [mm/h]	22	32	-	-
	CRP [mg/l]	4	3	-	-
	TNFαI therapy	ADA (Humira)			
**Patient 2**	Age [years]	32			
	Disease duration [years]	9			
	SW, n	9	5	-	-
	TEN, n	11	15	-	-
	VAS, [0-100 mm]	70	90	-	-
	DAS28-ESR	5.22	6.09	-	-
	ESR [mm/h]	9	18	-	-
	CRP [mg/l]	2	3	-	-
	TNFαI therapy	ADA (Humira)			
		**RA Patients With Secondary Loss Of Response To TNFαI Therapy (n = 3)**			
**Patient 3**	Age [years]	62			
	Disease duration [years]	5			
	SW, n	10	3	11	-
	TEN, n	14	8	13	-
	VAS, [0-100 mm]	60	45	65	-
	DAS28-ESR	6.68	4.38	6.03	-
	ESR [mm/h]	59	11	22	-
	CRP [mg/l]	15	11	10	-
	TNFαI therapy	ETA (Enbrel)			
**Patient 4**	Age [years]	60			-
	Disease duration [years]	21			-
	SW, n	6	3	10	-
	TEN, n	9	7	10	-
	VAS, [0-100 mm]	90	50	50	-
	DAS28-ESR	6.47	3.92	5.96	-
	ESR [mm/h]	57	6	41	-
	CRP [mg/l]	30	2	48	-
	TNFαI therapy	ETA (Enbrel)			
**Patient 5**	Age [years]	52			-
	Disease duration [years]	19			-
	SW, n	10	1	8	-
	TEN, n	10	0	12	-
	VAS, [0-100 mm]	80	5	70	-
	DAS28-ESR	5.73	1.48	5.57	-
	ESR [mm/h]	16	5	14	-
	CRP [mg/l]	40	2	2	-
	TNFαI therapy	ADA (Humira)			

CRP, C-reactive protein; DAS28-ESR, 28 joint disease activity score based on erythrocyte sedimentation rate; ESR, erythrocyte sedimentation rate; RA, rheumatoid arthritis; SW, swollen joint count; TEN, tender joint count; TNFαI, tumor necrosis factor-α inhibitors; VAS, visual analogue scale.
